# Lumbar Facet Joint Disease: What, Why, and When?

**DOI:** 10.3390/life14111480

**Published:** 2024-11-14

**Authors:** Wout Van Oosterwyck, Pieter Vander Cruyssen, Frédéric Castille, Erik Van de Kelft, Veronique Decaigny

**Affiliations:** 1 Department of Anaesthesia, Intensive Care and Pain Medicine, General Hospital Maria Middelares, 9000 Ghent, Belgium; 2 Department of Neurosurgery, Vitaz Hospital, 9100 St-Niklaas, Belgium; 3 Faculty of Medicine and Health Sciences, University of Antwerp, 2000 Antwerpen, Belgium

**Keywords:** lumbar facet joint, chronic low back pain, mechanical back pain, facetogenic low back pain, medial branch block, radiofrequency denervation, medial branch neurotomy

## Abstract

Low back pain (LBP) affects over 60% of individuals in their lifetime and is a leading cause of disability and increased healthcare expenditure. Facet joint pain (FJP) occurs in 27% to 40% of LBP patients but is often overlooked or misdiagnosed. Additionally, there is no clear correlation between the clinical examination, radiological findings, and clinical presentation, complicating the diagnosis and treatment of FJP. This narrative review aims to provide an overview of the literature regarding facet joint pain and discusses the utility of medial branch blocks (MBBs) and intra-articular (IA) injections as diagnostic and therapeutic tools prior to radiofrequency ablation (RFA). RFA is considered the gold standard for managing FJP, employing techniques that include precise needle placement and stimulation parameters to disrupt pain signals. Promising alternatives such as cooled RFA and cryodenervation require further research on their long-term efficacy and safety. Endoscopic denervation and multifidus stimulation are emerging therapies that may benefit chronic LBP patients, but additional research is needed to establish their effectiveness. When conservative management fails, RFA provides significant and lasting relief in well-selected patients and has a favourable safety profile. The current literature does not support surgical interventions for FJP management.

## 1. Introduction

Low back pain (LBP) affects over 60% of people during their lives, impacting nearly one in ten annually [1,2,3,4]. It can lead to significant daily life implications, including work absenteeism and a reduced quality of life [5]. Misdiagnosis of conditions like facet joint pain (FJP) is common, often resulting in ineffective treatments and unnecessary healthcare resource consumption [6,7]. The complex anatomy of the spine, combined with the lack of clear biomarkers from radiological findings or diagnostic tests, complicates an accurate diagnosis and treatment [1,8,9,10,11,12]. Although imaging methods like computed tomography (CT) and magnetic resonance imaging (MRI) are used, they do not consistently correlate with the clinical symptoms [13,14,15]. Consequently, effective management of LBP and FJP remains challenging, necessitating more precise diagnostic techniques and treatment approaches to address this prevalent issue [16,17].

This narrative review provides an overview of the relevant existing literature on LBP and FJP. It evaluates current diagnostic tools, such as imaging techniques, and their effectiveness in accurately identifying pain sources. Additionally, it examines available interventions, including medial branch blocks (MBBs) and radiofrequency ablation (RFA), assessing their efficacy and roles in pain management. Through this comprehensive analysis, the review aims to highlight gaps in knowledge and suggest future directions for research and practice.

RFA is regarded as the gold standard for interventional treatment for FJP, supported by the most robust evidence in the literature [2,11,16,18,19,20,21]. Its effectiveness is significantly enhanced when performed after MBBs, which help confirm the diagnosis and predict treatment outcomes [22,23]. By ensuring accurate targeting of the medial branches (MBs), RFA can provide substantial pain relief and improve function in well-selected patients, solidifying its role in managing chronic LBP.

## 2. Clinical Presentation

### 2.1. Clinical Presentation

LBP is a symptom of multiple potential pain generators, such as disc degeneration, facet joint disease, or spinal stenosis [24]. The clinical diagnosis of facetogenic pain is therefore challenging, as it is not easily distinguished from pain caused by other lower back structures [25,26]. In addition to spinal causes, the pain may also indicate a visceral pathology or mood disorder [6]. Due to the difficulty in identifying a precise source, facetogenic pain is often categorized as non-specific low back pain [26].

The pain is located in the paravertebral region of the lower back and presents uni- or bilaterally with possible radiation over the gluteal area, the groin, or thighs [11,16]. The pain in the low back is more prominent than the radiating pain to the leg, there is often no clear dermatomal pattern present, and no muscle weakness is reported in patients with facetogenic LBP, in contrast to patients with radicular pain [27]. Hence, the term pseudo-radicular pain is used [11]. Facetogenic pain typically does not extend beyond the knee. If pain radiates further, it is more likely due to a non-facetogenic cause, except in cases involving facet joint cysts where the cyst compresses the nerve root. In rare instances, facetogenic pain may also present as pelvic or abdominal discomfort [28].

Concerning the pain intensity, complaints are more pronounced with short-lived stiffness in the morning or after periods of immobilization. Light exercises, such as walking or gentle stretching, can help reduce the pain intensity. However, more strenuous activities tend to exacerbate the pain [29].

**Figure 1:**
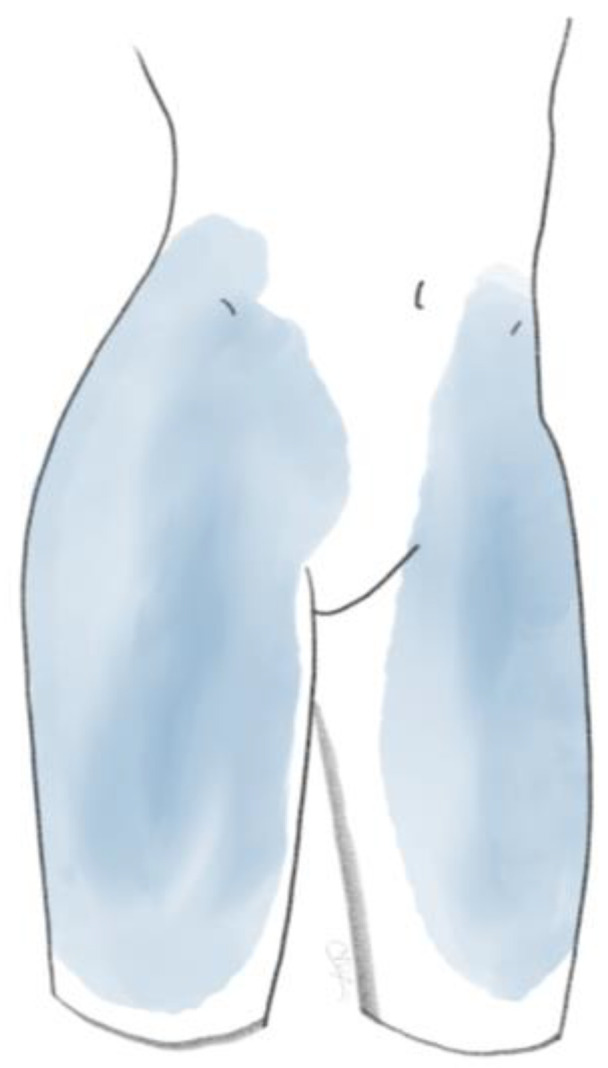
Anterior view of FJP.

**Figure 2:**
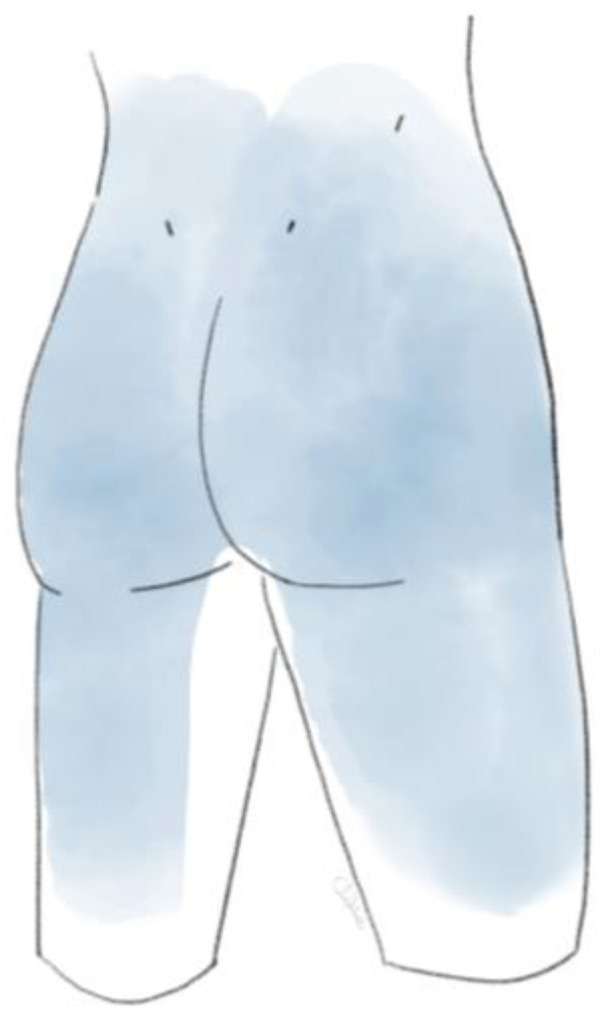
Posterior view of FJP.

Fukui et al. described particular pain patterns for the referred pain after applying provocation tests to specific individual facet joints [30]. The L1-L2 facet joint refers to pain in the lumbar spinal region. In addition, the L2-L3 facet joint rarely projects to the trochanteric, gluteal, and lateral thigh regions. The L3-L4 facet joint more prominently projects to the aforementioned areas, as well as the groin area less frequently. The L4-L5 facet joint refers to the lumbar spine and gluteal, lateral, posterior thigh, trochanteric, and groin areas. The L5-S1 facet joint refers to the same pain pattern, with the exception of the lumbar spine [30]. Even with the specific patterns known or proven, the location of pain is predominantly a clue rather than a direct correlation to a specific level [26]. Practically, the pain distribution could be described as a region covered by cycling shorts (Figure 1 and Figure 2).

Facet joint disease may lead to synovial cyst formation, which can act as space-occupying lesions. The cysts may compress nerve roots or the dorsal root ganglia (DRGs) and possibly create stenosis in the spinal canal. These symptoms resemble radicular signs, similar to those in disc herniation or spinal canal stenosis. A clearer dermatomal spread, as well as potentially radicular pain protruding in the lower limb is seen, which reaches beyond the knee, potentially to the foot and toes [31].

### 2.2. Clinical Examination

Diagnosing the cause of LBP is often complex due to its multifactorial nature and concurrent medical conditions. Although a proper clinical examination plays a significant role in the diagnosis of many diseases, it has not been proven to objectify facetogenic pain, even though many clinicians have tried.

Revel et al. proposed seven criteria, corresponding to a higher success rate of intra-articular facet joint infiltration [32]. However, these criteria were not consistently found by other researchers [33].

Stuber et al. found a limited negative predictive value for Kemp’s test. This pain provocation test is expected to elicit facetogenic or discogenic pain and involves backward flexion or extension simultaneously with the rotation of the upper body [12].

Paravertebral tenderness and pain on palpation over the facet joints can be found, although this is found to correlate only poorly with the response to facet joint treatment [16].

Unless nerve root or DRG compression is present due to facet joint cysts, the straight leg test is negative [34].

There are no abnormalities in muscle strength or reflexes [27].

### 2.3. Differential Diagnosis

For patients presenting with LBP, a differential diagnosis begins with excluding serious conditions like a systemic disease or conditions that affect the lumbar spine specifically [29,35,36]. Such conditions include compression fractures, spondyloarthropathy, malignancy, epidural abscesses, and cauda equina syndrome, as well as cases of radicular pain or spinal stenosis. These conditions require specific interventions and are distinct from non-specific LBP, where diagnostic tests do not reliably attribute pain to any single lumbar structure.

In cases of non-specific LBP, possible mechanical pain sources include the intervertebral disc, facet joints, sacroiliac joint, vertebral body, or associated muscles, fascia, and ligaments [29,36,37]. Intervertebral disc pain often involves midline tenderness and worsens with sitting, while facet joint pain typically presents as axial low back pain with possible referral to the hip or thigh. Sacroiliac joint pain can radiate into the buttock or leg and is often worsened by sitting or rising. Muscular pain, typically from repetitive strain, presents as axial pain with muscle spasm. Vertebral body pain, often related to compression fractures, presents as tenderness worsened by activity.

Advanced imaging, like MRI or CT, may be considered for specific conditions (e.g., suspected fractures or severe degeneration).

## 3. Epidemiology

LBP affects over 60% of individuals during their lifetime and impacts nearly one in ten people annually [1,2,3,4]. In 50% of patients, the pain subsides spontaneously within two weeks, and in 90%, it resolves within 12 weeks. Despite this, LBP remains the leading cause of years lived with disability. It is often only treated when it affects mobility and may otherwise be overlooked or dismissed [9].

Persistent pain lasting three to six months or longer leads to work absenteeism, a diminished quality of life, and increased healthcare costs [5]. It can result in social isolation, limitations in daily activity, chronic pain, and extended withdrawal from the workforce [5]. Several factors, including medication overuse, excessive healthcare resource consumption in search of a diagnosis, and unwarranted interventions (minimally invasive or surgical), further increase the cost and burden of LBP [6,7].

Quantifying the societal cost of LBP is difficult. In 2016, the costs related to neck and lower back pain in the U.S. were estimated to surpass USD 130 billion, primarily due to lost labour and increasing healthcare expenses [4]. In 2007, the annual healthcare costs associated with LBP were estimated at ANG 3.9 billion in the Netherlands and over EUR 50 billion in Germany [11].

FJP is estimated to occur in 27% to 40% of patients with LBP [11,16,25,34]. Despite being a leading cause of LBP, FJP is often misunderstood, leading to frequent misdiagnoses or inappropriate treatment. In FJP patients, osteoarthritis of the facet joint is the most frequent problem, with an estimated incidence in the overall adult population of 10 to 15% and a higher incidence in the more senior population [11,38].

## 4. Anatomy and Biomechanics

### 4.1. Anatomy and Pathological Changes of the Facet Joint

Understanding the anatomy and pathology of lumbar facet joints (LFJs) is essential for diagnosing low back pain and related conditions. LFJs are paired joints formed by the inferior articular process (IAP) of the vertebra above and the superior articular process (SAP) from the level below. They connect adjacent vertebrae, enabling movement and stabilizing the spinal column [8]. These synovial joints contain poorly vascularized hyaline cartilage, which inhibits proper healing after injury.

The capsule, consisting of two layers, has a volume of approximately 1 cc due to its recesses. Additionally, the capsule is rich in sensory nerve endings. These encapsulated and free nerve endings can be activated by injury or inflammation, leading to pain [39,40]. Inflammation typically results from mechanical stress, joint instability, or the degeneration of nearby tissues, with pro-inflammatory cytokines like TNF-α, IL-1, and IL-6, as well as Substance P, playing key roles in the pain-generating mechanisms, correlating with mechanisms seen in rheumatoid arthritis [2,39,40].

### 4.2. Biomechanics of the Lower Vertebral Element

Biomechanically, both LFJ together with the intervertebral discs form a three-joint complex, where the LFJs are essential for stabilizing the spine during movement [8]. The surface of the IAP is convex, whereas the SAP is concave. Each facet joint bears a notable portion of the axial load, carrying 10 to 20% in physiological circumstances. Particularly when the intervertebral discs are compromised or dehydrated, the stress on the joints increases and leads to degeneration, which is especially prevalent at levels L4-L5 and L5-S1 [8,16,26,34,39]. During hyperextension or cyclic flexion movements, significant strain can occur across the LFJ capsules, since they are shorter and more tough than the cervical facet joints. This strain can potentially result in fibrocartilaginous metaplasia and increased laxity of the joints [26,39]. This creates a complex interaction where the degeneration in one facet joint can influence the biomechanics and integrity of the entire spinal segment, further exacerbating pain and dysfunction [8,9].

Variations in the lumbar spine, such as sacralization or lumbarization, are common in the general population and may complicate surgical interventions. A retrospective analysis found a 9.9% prevalence of lumbosacral transitional vertebrae (LSTVs), with lumbarized S1 occurring in 5.8% and sacralized L5 in 4.1% of cases. Of the LSTV cases, 69.6% were not identified by the reporting radiologist, and occasional misreporting between sacralization and lumbarization occurred [11,41].

In case of asymmetry in the orientation of the articular planes of the LFJs at the same level, the terminology tropism is used. Misalignment of up to 5 or 10% is considered normal, although a formal consensus is lacking [8]. It was hypothesized that tropism would result in or accelerate osteoarthritis (OA) due to an imbalance of forces. This was not found in cadaver studies nor in studies evaluating facet joint orientation and tropism on CT scans in different study populations [10,13].

### 4.3. Anatomy of the Nerves

The medial branch (MB) of the dorsal ramus plays a crucial role in innervating the facet joints and adjacent structures, including the multifidi muscles, interspinous ligament, and supraspinous ligament [42]. It is the sole supplier of motor fibres to the multifidus muscles, while also providing sensory input to the facet joints and ligaments. The MB courses along the lateral neck of the SAP in the groove formed by the SAP and the transverse process, descending caudally and posteriorly, often accompanied by vessels from the lumbar artery and vein [8,42,43]. In middle two quarters of the neck, the MB adheres to the periosteum and is well exposed and susceptible to denervation for a limited length [44,45]. Before entering the fibro-osseous canal, the nerve gives rise to small articular branches that innervate the lateral and inferior aspects of the LFJ. The nerve’s position near the mamilloaccessory ligament offers a relatively predictable target for MB neurotomy. However, variations in the anatomy of the MB can complicate accurate electrode placement, especially at different points along the SAP.

The L1-L3 MBs descend 1–2 vertebrae, while the L4-L5 MBs descend 2–3 vertebrae, extending their innervation to the lumbosacral joint and the dorsal aspect of the sacrum [42]. These branches form a network with the adjacent medial and lateral branches, as well as the dorsal rami, creating a complex system of nerve connections.

Research in rats indicates that labelled neurons from the L5-L6 facet joints originate in ipsilateral DRGs from L1 to L5 and in paravertebral sympathetic ganglia from T12 to L6, highlighting the intricate relationship between the sensory and sympathetic systems. Some sensory fibres from the facet joints may even pass through the paravertebral sympathetic trunk and reach the L1 or L2 DRGs, which could explain referred pain patterns, such as inguinal or anterior thigh pain in patients with lower LFJ lesions [46].

Repeated trauma or injury to the spine and facet joints can lead to the compression or retraction of the medial and lateral branches, resulting in chronic low back pain [42]. Additionally, the close association of the MB with the posterior branch of the lumbar artery increases the risk of minor complications during repeated interventions, such as MB neurotomy, potentially leading to vascular injury or hematoma formation.

Despite the anatomical consistency in some areas, such as the MB’s course through the mamilloaccessory notch, variability in other regions and the presence of complex neural plexuses, including connections with the intermediate branch, complicate the diagnosis and treatment. Precision in targeting the medial branch is crucial, as improper needle placement during neurotomy can inadvertently affect the lateral or intermediate branches [44].

## 5. Diagnostic Interventions

There is no clear consensus on how to best evaluate LFJ OA with imaging. Imaging often does not correlate well with clinical symptoms of LBP, and its primary utility may lie in ruling out more severe conditions (red flags) rather than identifying symptomatic facet joint arthritis [1,11,15]. Despite extensive imaging findings in asymptomatic individuals, such as disc degeneration or facet joint pathology, these findings often lack specificity for diagnosing the source of LBP [2,10,47].

### 5.1. X-Ray

Oblique radiographs provide the best view of LFJs, offering a “Scottie dog” view, which is useful for identifying degenerative changes. However, X-rays are limited in providing detailed information on facet joint degeneration.

### 5.2. CT

CT scans improve the anatomical evaluation of facet joints and are considered the preferred method for imaging LFJ OA. They reveal joint space narrowing, sclerosis, subchondral erosions, and osteophyte formation, among other degenerative signs. CT scans show a high prevalence of facet joint degeneration, but the relationship between these findings and actual pain is weak [10].

### 5.3. MRI

MRI is highly sensitive and specific for detecting facet joint degeneration, subchondral oedema, and surrounding neural impingement. However, studies have shown inconsistent correlations between MRI findings and clinical symptoms, with some degenerative changes found in patients without pain [48,49]. MRI has a slight advantage over CT in evaluating neural impingement but may underestimate the severity of LFJ OA compared to CT. Additionally, MRI does not use potential harmful radiation [50].

### 5.4. Single-Photon Emission Computed Tomography-CT (SPECT-CT)

**Figure 3:**
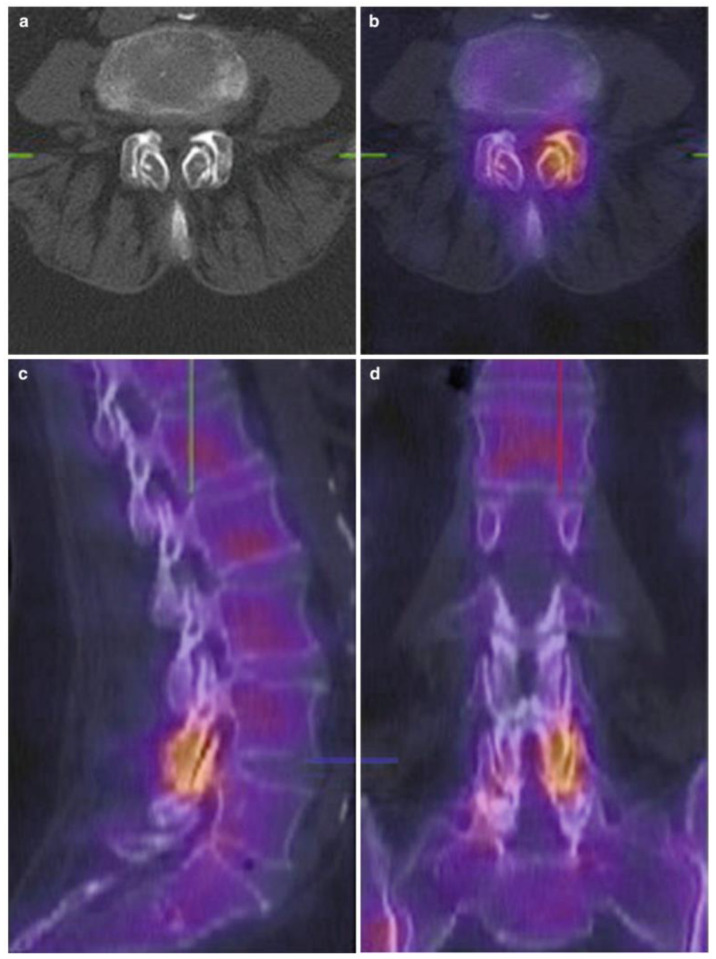
SPECT-CT of the lumbar spine. Woman with low back pain and a typical presentation of facet joint osteoarthritis on SPECT/CT at the L4–L5 level. Axial CT image (**a**) and axial (**b**), sagittal (**c**), and coronal (**d**) SPECT/CT images. Courtesy by E.V.d.Kelft.

SPECT-CT is recognized for its potential in diagnosing LFJ disease. It can detect areas of increased metabolic activity or “hot spots” that may indicate active inflammation in the facet joints, helping identify pain sources more accurately. Studies show that pathological findings are present in 86% of lumbar SPECT-CT scans, although not all detected abnormalities correlate with pain. Approximately 65% of these identified areas are linked to facet joint pain [51] (Figure 3).

Research indicates that patients with SPECT-positive scans are more likely to respond to MBBs [52]. This suggests that SPECT-CT may help predict outcomes following treatment. Additionally, patients with SPECT-positive scans experienced greater reductions in pain scores at one month of follow-up [53].

A notable advantage of SPECT-CT is its ability to provide a whole-body map of metabolic activity with the occurrence of hot spots of high activity. These hot spots are significantly more frequently found in the lumbar spine in the presence of LBP [14].

Although SPECT-CT shows promise, its routine clinical use remains limited due to concerns about specificity, as some patients with positive imaging findings may not experience pain. Moreover, further research is needed to assess its cost-effectiveness and refine its role in diagnosing lumbar facet joint disease [9,21].

### 5.5. Intra-Articular Infiltration

Intra-articular (IA) injections target the facet joint itself, injecting a combination of a local anaesthetic and possibly a corticosteroid directly into the joint space. These injections are used both for diagnostic purposes and for therapeutic management of facet joint-mediated LBP. However, like MBBs, their efficacy as a long-term treatment has not been proven. Cohen et al. even suggested that IA infiltrations should not be considered therapeutic, given the absence of long-term effects [2,23].

IA injections can be used diagnostically to confirm that a specific facet joint is the source of pain. If the injection significantly reduces pain, it is assumed that the facet joint is contributing to the patient’s symptoms. However, compared to MBBs, IA injections are less commonly used purely for diagnostic purposes, given that many guidelines recommend the usage of MBBs [19,23].

The diagnostic accuracy of IA injections can also be compromised by the spread of the injectate beyond the joint capsule, potentially anesthetizing other structures and leading to false-positive results. IA injections are technically more challenging to perform than MBBs due to the difficulty of accurately entering the small joint space. This is particularly true in patients with advanced degenerative changes in the facet joints, where joint space narrowing and osteophyte formation can make needle placement more difficult. Failure rates can reach up to 29% [23]. However, pericapsular injection was found to be as effective as proper intracapsular injection [23,54]. As with MBBs, fluoroscopic or ultrasound guidance is typically applied to ensure proper needle placement and avoid intravascular uptake.

Steroid injections play a dual role in managing OA pain, including LFJ OA, by reducing inflammation and interrupting nociceptive inputs at both the central and peripheral levels. However, their long-term efficacy remains questionable, and they are often used as part of a broader treatment strategy that may include physical therapy, medications, and other interventions [2].

In terms of risks, IA injections carry similar complications to MBBs, such as infection, bleeding, and injury to surrounding structures. Additionally, the use of corticosteroids can lead to potential side effects, including localized tissue atrophy and systemic effects such as elevated blood sugar levels in diabetic patients.

### 5.6. Medial Branch Blocks

MBBs are widely used as a diagnostic tool to identify facet joint-mediated pain and as a prognostic indicator for the success of subsequent RFA. However, their diagnostic accuracy is subject to limitations, including high false-positive rates, which range from 25% to 40% [55,56]. This variability is influenced by factors such as placebo effects, the spread of the injectate to other pain-generating structures, and the excessive use of local anaesthetic [57]. False-negative MBBs have been less studied but remain a concern. Vascular uptake during the procedure, reported in 6% to 30% of cases, and aberrant innervation are potential causes of false negatives [26,42,58,59]. Intravascular uptake can often be missed, making it essential to confirm with contrast imaging, despite the increased radiation exposure.

The technique of performing MBBs also varies widely. International consensus guidelines advocate for using ultrasound or fluoroscopic guidance and smaller volumes of local anaesthetic (0.25–0.5 mL) to prevent the spread to adjacent structures and increase diagnostic accuracy [57,58,60]. Larger volumes or inadvertent spread to other structures, such as the intervertebral foramina or posterior muscles, can lead to misdiagnosis.

There is no clear consensus on how to define a “positive” block [20,22,61]. While some authors argue that 50% pain relief should be the threshold, others suggest a stricter criterion of 70% to 80% pain relief. A confirmatory second block is often recommended to reduce the false-positive rate, which can reach up to 45% [23]. Studies show that patients with 80% or more pain relief after the first MBB are significantly more likely to achieve similar relief after a second block [60]. Therefore, some guidelines recommend performing two diagnostic MBBs and requiring at least 80% pain relief to confirm a diagnosis [18,19]. However, even with stringent criteria, anatomical variations and aberrant innervation complicate diagnosis.

Studies also highlight the therapeutic potential of MBBs, with some patients experiencing prolonged pain relief, though the mechanisms behind this are not fully understood [62,63]. Despite this, most rigorous studies have not supported the routine use of MBBs as a therapeutic intervention, and their primary role remains diagnostic.

Cost-effectiveness studies, such as the one conducted by Cohen et al., offer insights into the financial considerations of using MBBs [64]. In a randomized comparative study of 151 patients, the effectiveness and cost of zero, one, or two diagnostic MBBs prior to RFA were evaluated. The group with no diagnostic MBBs had a 33% success rate at a cost of USD 6286 per successful outcome. In contrast, the group that received one diagnostic MBB had a lower success rate of 16% at USD 17,142, while the group that received two MBBs had a 22% success rate at USD 15,241. These findings highlight the cost–benefit trade-offs associated with using multiple diagnostic MBBs before RFA.

Given the high costs and variability in outcomes, cost-effectiveness analyses suggest that a single set of MBBs with at least 50% pain relief may be sufficient in clinical practice. However, for more stringent scenarios, such as clinical trials, two sets of blocks may be necessary to ensure the highest likelihood of successful outcomes of RFA [19,21].

## 6. Therapeutic Interventions

### 6.1. Conservative Medical Management

Conservative management of chronic low back pain involves a comprehensive approach that integrates both medication and physical therapy. Pain relief may be sought through short-term use of painkillers like paracetamol or non-steroidal anti-inflammatory medications (NSAIDs), though these offer limited long-term benefits and must be used cautiously due to potential side effects [1,16,47,65]. Physical therapy is equally critical, focusing on core strengthening, reducing lumbar lordosis with pelvic tilting, and incorporating aerobic exercises. A Cochrane review conducted in 2021 found a clinical improvement in the short and intermediate terms. The results on long-term improvements were inconclusive [66].

According to the National Institute for Health and Care Excellence (NICE) guidelines, patients should be encouraged to continue their normal activities and engage in group exercise programs that combine biomechanical, aerobic, and mind–body approaches for optimal recovery [67].

Manual therapy, such as spinal manipulation or soft tissue techniques, is advised only as part of a broader treatment plan that includes exercise and possibly psychological support. The goal is to promote a return to daily activities, including work, while avoiding specific interventions like belts, foot orthotics, acupuncture, or electrical nerve stimulation, which have not shown long-term efficacy [67].

In cases where NSAIDs are unsuitable or ineffective, weak opioids may be considered for acute low back pain. A Cochrane review from 2023 found short-term benefits from opioids for chronic LBP, but unwanted side effects often arise [68]. Medications like selective serotonin reuptake inhibitors (SSRIs), tricyclic antidepressants (TCAs), gabapentinoids, or antiepileptics are not appropriate for managing LBP, as is paracetamol as a monotherapy [32]. There is some evidence for the use of duloxetine, a serotonin and norepinephrine reuptake inhibitor (SNRI), in chronic LBP, but this is not incorporated in the NICE guidelines [47,69]. For a more holistic approach, patient education, self-care, and continued participation in normal daily routines, combined with tailored physical therapy, are key elements in improving pain management outcomes [66].

### 6.2. Intra-Articular Injections

Injections like IA and MBBs are commonly used to treat lumbar facet joint pain, but their therapeutic effectiveness has been inconsistent [16,23,70]. Comparisons between IA injections and placebo treatments have shown no significant long-term benefit. IA injections did not provide lasting pain relief or delay the need for RFA [71]. Thus, clinical evidence does not support the widespread use of IA injections [72]. As a result, several medical societies recommend against routine IA procedures due to their limited efficacy in managing FJP [18,19,22,73].

An alternative gaining attention is platelet-rich plasma (PRP) therapy, which uses a patient’s own blood components to promote healing and reduce inflammation. Some studies suggest that PRP injections may be more effective than traditional steroid-based injections for treating FJP [74,75]. However, further research is needed to confirm its long-term effectiveness and broader applicability.

### 6.3. Radiofrequency Ablation

RFA or radiofrequency denervation is a procedure used to alleviate chronic pain by applying radiofrequency (RF) energy to targeted nerves. This technique encompasses both continuous and pulsed RF. Continuous RF generates an electromagnetic field that causes ionic agitation in the tissues surrounding the needle’s tip, producing heat that disrupts nerve function through denaturation and thereby blocking pain signals [11,65,76]. In contrast, pulsed RF creates short but intense electromagnetic fields that minimize damage to surrounding tissues by maintaining a temperature below 42 °C [77]. The precise mechanism by which pulsed RF alleviates pain is not fully understood. Cooled RF, a variation of continuous RF, employs cold saline as a heatsink to create larger, controlled lesions, potentially allowing for more extensive pain relief [70].

The success of RFA is highly dependent on proper patient selection [11,16,60,78]. Diagnostic blocks are crucial for identifying patients with FJP who are most likely to benefit from the procedure. While RFA can offer temporary relief, the effects are not permanent, as the nerves eventually regenerate. Therefore, the procedure can be repeated, with a recommended limit of up to two treatments per year [11]. Although widely used, some studies have raised concerns about its long-term efficacy, suggesting that its benefits may be limited for certain patient groups [2,24,67].

### 6.4. Continuous Radiofrequency Ablation

Diagnostic MBBs, especially with dual blocks achieving 75% to 100% pain relief, have strong evidence supporting their use in identifying candidates for RFA. Lesser relief, around 50% to 74%, also shows fair evidence, but single diagnostic blocks provide more limited validation [79].

RFA is widely considered the “gold standard” for interventional treatment of facet joint-related LBP, with meta-analyses demonstrating significant pain reduction lasting up to 12 months [16,19,20,80]. Studies report a significant reduction in pain, typically a 2–3 point decrease on the visual analogue scale (VAS), compared to control groups [11].

#### 6.4.1. Technique

RFA for FJP requires precise needle placement near the MB [5,50,68]. Historically, a perpendicular approach was used, but a parallel needle placement is now widely accepted given the ellipsoid cauterization lesion rather than a spherical lesion at the tip. There is an international consensus that a longer lesion of the MB correlates to a longer duration of relief [16,18,20,81]. Advanced imaging is recommended, with fluoroscopy being the most commonly used, though ultrasound is an option. Sterile precautions, such as disinfecting the skin and wearing a mask, gown, and sterile gloves, are essential. The procedure can be performed with or without light sedation, though general anaesthesia is not routinely recommended. Local anaesthesia is often used to facilitate the introduction of RF needles.

The needle is guided toward the junction of the SAP and the transverse process, commonly referred to as the “eye of the Scotty dog”. The goal is to target the medial aspect of the lateral side of the SAP, with the position verified using anteroposterior, oblique, and lateral imaging, along with electrical stimulation [43]. Motor stimulation is strongly advised by multiple societies like the American Society of Regional Anesthesia (ASRA) to confirm proximity to the target nerve through contraction of the multifidus muscle while avoiding stimulation of the ventral ramus, which could cause a lower limb response. The International Pain and Spine Intervention Society (IPSIS) does not recommend sensory or motor stimulation due to the chance of a false feeling of safety and recommends fluoroscopic objectivation of the needle tip relative to the bony landmarks [82]. A safety threshold of 2 V is recommended for motor stimulation, while sensory stimulation should ideally be less than 0.5 V. Typically, RF is applied at 80 °C for 90–120 s, with the option to inject steroids afterward to reduce the chance of post-procedural inflammation and neuritis [19,21].

Patient-specific factors like age, comorbidities, and anatomy play critical roles in determining RF outcomes. Though sensory stimulation is widely used, studies show that it may not significantly impact long-term pain relief [83]. Other methods, such as targeting anatomical landmarks and using motor responses like paraspinal muscle contractions, have demonstrated positive results. Multiple lumbar levels are often treated simultaneously due to the challenge of pinpointing the exact facet joint responsible for the pain and because each facet joint is innervated by two medial branches.

#### 6.4.2. Orientation

**Figure 4:**
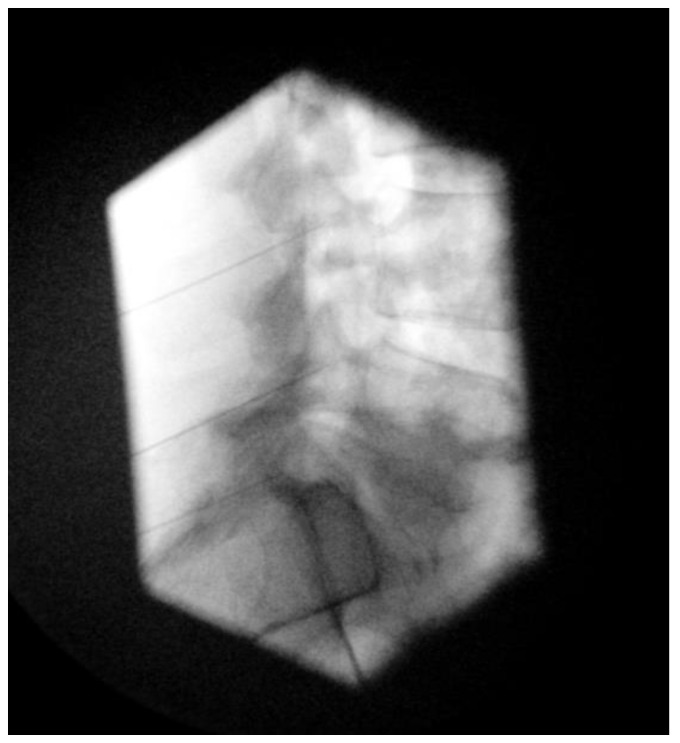
RFA needle placement. Lateral image. Inclination in relation to the top end plate of L4 24°, L5 40°, and sacrum 57°.

**Figure 5:**
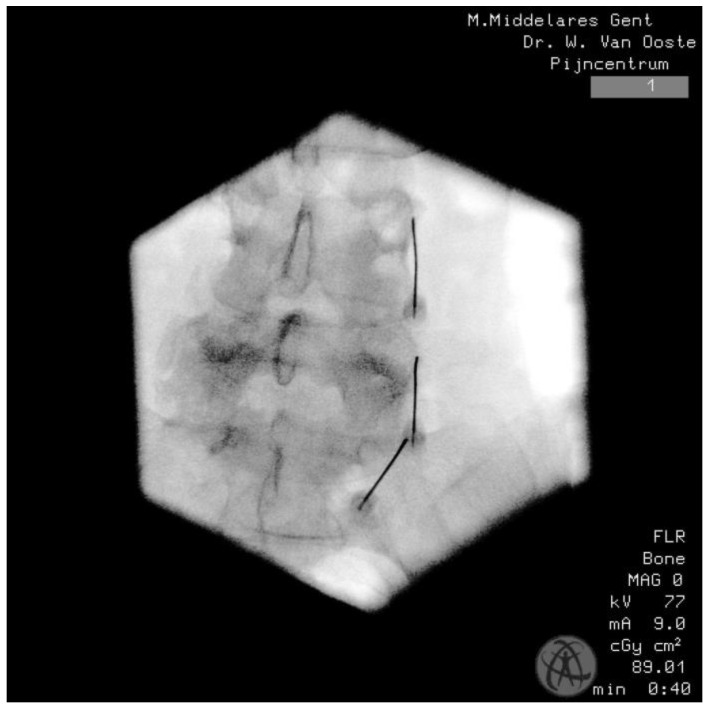
RFA needle placement. A 50-year old woman. Lateral 10° oblique.

The C-arm is typically positioned ipsilateral at a minimal oblique angle (10–20°) and in a caudad–cephalad direction. A recent study advocates for a more caudal angulation than was previously accepted, with caudal angulation reaching up to more than 40° from the cranial endplate of the designated vertebral level [84] (Figure 4 and Figure 5).

#### 6.4.3. Needle Characteristics and RF Settings

A wide range of needles is available. There is variation in the length of the active needle tip, ranging from 2 mm up to 15 mm. A longer active tip results in a longer lesion [62]. Most commonly used needles for MB denervation have an active tip of 5 to 10 mm [19,20,21].

The tip can either be straight, curved, or can be deployed when it is in the right location. Curved tips are considered more easily steerable and are able to follow the curvature of the SAP, resulting in a more pronounced overlap with the nerve. Some needles can be deployed or unfolded, resulting in a bigger lesion and thus increasing the chance of capturing the MB [19].

RF needles come in various sizes, with the more frequently used sizes ranging from 16 to 22 gauge. Bigger needles create bigger lesions but cause more procedural pain or discomfort.

When the temperature of the probe is increased, the heat will spread further, resulting in a bigger lesion. At high temperatures, charring and vaporization of the tissue can occur. This should be avoided. Therefore the recommended temperature is 80 to 90 °C [19,21].

Cosman et al. published an overview of different parameters and their effects on the lesion size. For example, they found that a 16 ga needle with a temperature set at 90 °C for 3 min resulted in a lesion of 11,1 mm in diameter, whereas a 18 ga needle with a temperature set at 80 °C for 2 min, resulted in a lesion of 7.6 mm in diameter [76].

#### 6.4.4. Complications

RFA is generally considered safe, though it does carry some risks [85]. Potential complications include infection, haemorrhage, increased pain, numbness, or dysesthesias. Most adverse events are mild and self-limiting, such as superficial infections, rashes, or vasovagal episodes. Serious complications, like neuraxial infections or nerve root irritation, are exceedingly rare. Considering infection, the risk from RFA may even be lower than that of diagnostic blocks due to the protective heat used during the procedure [23,64,79].

Muscle-related side effects, particularly involving the multifidus muscle, are a concern with RFA. Studies indicate that while multifidus dysfunction occurs post-procedure, it may be more severe than in other surgical interventions, such as posterior lumbar fusion, due to nerve damage rather than direct muscle injury [86,87,88]. Post-procedure neuritis, lasting several weeks, affects 1–10% of patients, and some may experience neuropathic pain over the lumbar paraspinal muscles [16]. Although multifidus atrophy after RFA has not been conclusively proven, it remains a plausible concern requiring further investigation.

### 6.5. Cooled Radiofrequency Ablation

Cooled radiofrequency ablation (C-RFA) has shown promising results in treating FJP, offering advantages over traditional RF techniques. By creating larger, spherical lesions, it allows for greater flexibility in probe placement and improves the likelihood of targeting nerves effectively, particularly in cases with anatomical variations. A study comparing C-RFA to IA infiltrations found that patients treated with C-RFA were more likely to experience significant pain relief. These findings suggest that C-RFA may be a more effective option for long-term pain management in well-selected patients [70].

### 6.6. Pulsed Radiofrequency Ablation

Multiple randomized trials and systematic reviews have shown that pulsed radiofrequency ablation (P-RFA) is less effective than conventional RFA for treating FJP [9]. P-RFA provides minimal benefits through 12 months and is less effective at reducing pain and improving function. As a result, P-RFA cannot replace conventional RFA for long-term pain management.

### 6.7. Chemical Denervation

Afifi et al. (2022) compared RFA of LFJ with chemical neurolysis using ethyl alcohol (EA-95) or glycerol (Gly-20) in 95 patients. The study found that RFA provided significantly better pain relief and quality of life improvements than both chemical agents. RFA-treated patients reported lower pain scores at 6 weeks, 6 months, and 1 year compared to those treated with EA-95 and Gly-20. In contrast, chemical neurolysis was associated with risks like tissue necrosis, neuritis, and uncontrolled diffusion, leading to complications like painful paraesthesia months after treatment [89].

### 6.8. Cryodenervation

Cryodenervation, developed in the 1970s, uses a cryoprobe to freeze nerves to −50 °C with medical-grade CO_2_, causing endoneural oedema and nerve cell death. This method, used for facet joint neurotomy, achieves successful outcomes in about 65–70% of cases [11,54]. Unlike RFA, cryodenervation does not require precise probe placement, although sensory stimulation is advisable. Peri-procedural pain is generally tolerable. While it has shown promising results, such as pain reduction at 6 weeks, 3 months, and 6 months, no direct comparative studies with RFA for FJP are available. Therefore, further research is needed to confirm its long-term effectiveness compared to other techniques [90].

### 6.9. Endoscopic Denervation

Endoscopic denervation (ED) is an advanced form of RFA targeting the MB, providing direct visualization of anatomical structures like the nerve root and articular capsule. This allows for more precise nerve detection and complete denervation, leading to stable and long-lasting pain relief [80,90,91]. Compared to traditional RFA, ED reduces the risk of nerve injury and sensory loss while offering more thorough ablation. However, the procedure is more surgical in nature, takes longer to perform, requires extended recovery time, and incurs higher costs. Despite these drawbacks, ED provides better and longer-lasting analgesia than percutaneous RFA.

Patients treated with ED saw their VAS scores drop substantially, with 58% of patients experiencing pain relief after six weeks and 38% experiencing sustained relief after twelve months [92]. ED has shown even greater efficacy for patients with isolated facet joint OA. Although some patients require re-operations due to recurring symptoms, the technique can be repeated without causing structural changes, making it a viable long-term treatment for chronic FJP [91].

## 7. Surgical Possibilities

### 7.1. Controversy Regarding Surgery and Other Invasive Procedures

The role of surgery in managing LBP is controversial, particularly when neural compression or a structural deformity is absent. NICE guidelines strongly advise against spinal fusion or disc replacement in LBP management, recommending these procedures only within controlled clinical trials [47]. Despite the lack of robust evidence, the number of elective lumbar spine surgeries, including fusions, has significantly increased, particularly for degenerative conditions like spondylolisthesis [93,94]. The effectiveness of procedures such as lumbar fusion remains debated, with some studies showing no clear advantage over conservative treatments like cognitive behavioural therapy [95]. Placebo effects and the natural course of LBP further complicate the assessment of surgery’s long-term efficacy [93].

### 7.2. Spine Surgery with Facet Arthroplasty

Facet arthroplasty, using devices like TOPS and FENIX, has been proposed as a treatment option for mechanical LBP, particularly in cases involving conditions like spondylolisthesis [96,97]. The TOPS device, designed for dynamic stabilization, showed an improvement in patient outcomes, including significant reductions in pain scores. However, it is crucial to note that the trials were primarily designed for patients with radicular symptoms, such as neurogenic claudication or radiculopathy, rather than for low back pain itself. As a result, its application in treating low back pain, especially when neural compression is not involved, remains controversial. The long-term benefits, particularly for non-radicular mechanical pain, are not well-established, and more focused studies are needed to confirm its role.

**Figure 6:**
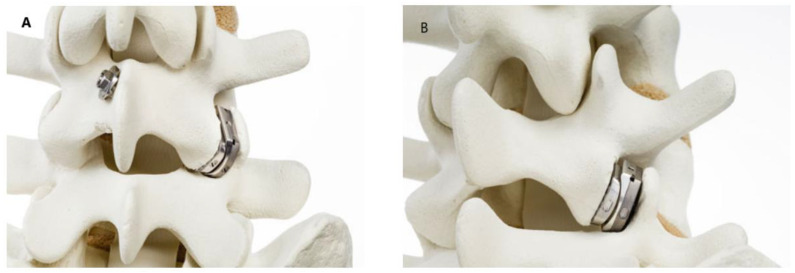
FENIX arthroplasty. (**A**) Detachment of the inserter; the implantation of FENIX^®^ is completed. (**B**) Note the perfect fit between the two implants, courtesy of E.V.d.K.

In contrast, the FENIX device, specifically designed for facet joint-related pain, has shown more targeted results. In a small proof-of-concept and feasibility study, patients experienced significant pain relief and functional improvements, with most maintaining full mobility at 24 months post-surgery. However, the limited size and scope of this trial imply that its outcomes, though encouraging, should be interpreted cautiously [96]. Broader, longer-term studies are necessary to substantiate its efficacy for treating FJP and confirm its safety profile, particularly in avoiding complications like adjacent segment disease (Figure 6).

Given the mixed evidence and limited scope of current studies, both the TOPS and FENIX devices should be considered experimental options. Their use for purely mechanical LBP remains unproven, and more rigorous trials specifically designed to address this condition are needed before their widespread adoption in clinical practice.

### 7.3. Multifidus Stimulation for Chronic Low Back Pain

Multifidus stimulation, using devices such as the ReActiv8 (Mainstay Medical, Dublin, Ireland), represents an emerging treatment for chronic mechanical LBP, particularly in patients who have not responded to conservative therapy, including RFA [94,98]. This restorative neurostimulation approach targets motor control deficits in the multifidus muscle, which are often implicated in chronic LBP; thus, the treatment is not specifically aimed at people suffering from facetogenic pain. Early studies suggest that patients can achieve significant improvements in their LBP, with some experiencing a 50% reduction in pain scores and improved disability outcomes over three years of treatment [99]. However, while the ReActiv8 device shows promise, especially for patients with long-standing, refractory LBP, it remains an evolving therapy.

Despite these promising results, caution is warranted, as its long-term effectiveness and its comparative efficacy with other treatments like RFA remain unclear. Further research is needed to determine its role within the larger spectrum of LBP management, especially considering the mixed success of neuromodulation therapies in this field.

## 8. Summary

Low back pain (LBP) affects about 10% of individuals yearly and is a leading cause of disability and healthcare resource consumption globally [4,11]. Facet joint pain (FJP) occurs in 27% to 40% of LBP patients, complicating the diagnosis due to overlapping symptoms with conditions like disc degeneration and stenosis [6,9,25]. Both clinical examinations and radiological studies demonstrate limited predictive value for diagnosing FJP [26].

The lumbar facet joints (LFJs) are synovial joints that form a three-joint complex with intervertebral discs [8,40]. Degeneration of a facet joint or an intervertebral disc can exacerbate pain and dysfunction across the spinal segment [9,16]. Conservative management typically involves short-term NSAIDs and physical therapy, while strong opioids are not recommended for chronic LBP [65,66,67].

Medial branch blocks (MBBs) are frequently used to diagnose facet joint-mediated pain and predict the success of radiofrequency ablation (RFA) [18,22,47]. However, they have high false-positive rates (25–40%) and varying accuracy, fuelling the debate on whether zero, one or two MBBs are ideal [19,22,23,79]. There is insufficient evidence to support the routine use of intra-articular injections for FJP [23,70,71,72].

RFA is recognized as the gold standard for interventional treatment for FJP, utilizing heat to disrupt nerve function and block pain signals [2,9,11,16,19,80]. The success of RFA is contingent upon precise needle placement near the medial branch, with a low complication rate [64,84,85]. Alternatives like cooled-RFA, cryodenervation, and endoscopic denervation show promise, offering enhanced targeting and pain relief, while pulsed-RFA has proven less effective [9,54,70,80,90]. The current literature lacks evidence for surgical options such as spinal fusion outside of controlled trials for facetogenic low back pain. Further research is needed to validate emerging treatments like multifidus stimulation [11,47,93,94].

## Data Availability

No new data were created or analyzed in this study. Data sharing is not applicable to this article.

## Abbreviations

ASRA	American Society of Regional Anesthesia
C-RFA	cooled radiofrequency ablation
CT	computed tomography
DRG	dorsal root ganglion
ED	endoscopic denervation
FJP	facet joint pain
IA	intra-articular
IAP	inferior articular process
IPSIS	International Pain and Spine Intervention Society
LFJ	lumbar facet joint
LPB	low back pain
LSTV	lumbosacral transitional vertebrae
MB	medial branch
MBB	medial branch block
MRI	magnetic resonance imaging (MRI)
NICE	National Institute for Health and Care Excellence
NSAID	non-steroidal anti-inflammatory medication
OA	osteoarthritis
P-RFA	pulsed radiofrequency ablation
PRP	platelet-rich plasma
RF	radiofrequency
RFA	radiofrequency ablation
SAP	superior articular process
SNRI	serotonin and norepinephrine reuptake inhibitors
SPECT-CT	single-photon emission computed tomography-CT
SSRI	selective serotonin reuptake inhibitor
TCA	tricyclic antidepressant
VAS	visual analogue scale
